# LncRNA FOXD1‐AS1 acts as a potential oncogenic biomarker in glioma

**DOI:** 10.1111/cns.13152

**Published:** 2019-05-17

**Authors:** Yuan‐Feng Gao, Jun‐Yan Liu, Xiao‐Yuan Mao, Zheng‐Wen He, Tao Zhu, Zhi‐Bin Wang, Xi Li, Ji‐Ye Yin, Wei Zhang, Hong‐Hao Zhou, Zhao‐Qian Liu

**Affiliations:** ^1^ Department of Clinical Pharmacology, Xiangya Hospital Central South University Changsha China; ^2^ Hunan Key Laboratory of Pharmacogenetics, Institute of Clinical Pharmacology Central South University Changsha China; ^3^ Department of Pharmacy The First Hospital of Hunan University of Chinese Medicine Changsha China; ^4^ Department of Orthopaedics The First Affiliated Hospital of the University of South China Hengyang China; ^5^ Department of Neurosurgery, The Affiliated Cancer Hospital of XiangYa School of Medicine Central South University Changsha China

**Keywords:** eIF5a, glioma, lncRNA FOXD1‐AS1, lncRNAs, miR‐339/342

## Abstract

**Aims:**

Altered activities of long noncoding RNAs (lncRNAs) have been associated with cancer development, and lncRNA FOXD1‐AS1 (FOXD1‐AS1) is the antisense transcript of the gene encoding for FOXD1, known for its role as an oncogene in several tumor types including glioma. However, the role of FOXD1‐AS1 in the differentiation and progression of glioma is not well known.

**Methods:**

Expression profile chip and qPCR were used to screen and identify FOXD1‐AS1. Glioma cells were transfected with siRNA or eukaryotic expression vector to observe FOXD1‐AS1 function in vitro and in vivo. Dual luciferase reporter gene analysis, Western blot, and ChIRP‐MS were used to detect microRNAs and protein that combine with FOXD1‐AS1.

**Results:**

FOXD1‐AS1 was upregulated and directly correlated with the glioma grade, and it was localized in both the nucleus and the cytoplasm of the glioma cell. FOXD1‐AS1 silencing caused tumor suppressive effects via inhibiting cell proliferation, migration, and apoptosis, while FOXD1‐AS1 overexpression resulted in opposite effects. Additionally, in vivo experiments showed that FOXD1‐AS1 knockdown reduced tumor volume and weight. More importantly, mechanical studies revealed that FOXD1‐AS1 targeted both miR339‐5p and miR342‐3p (miR339/342). Furthermore, protein eukaryotic translation initiation factor 5 subunit A (eIF5a) resulted a direct target of FOXD1‐AS1.

**Conclusions:**

These data indicated that FOXD1‐AS1, a miR339/342 target, affected biological processes via protein eIF5a; thus, it might be considered as a new therapeutic target for glioblastoma.

## 1 INTRODUCTION

Glioma is a common malignant tumor affecting the brain and/or the spine, accounting for approximately 40% of the intracranial tumors.^1^, ^2^ The WHO classifies gliomas into four levels, where grade I is a benign glioma, II is a low‐grade glioma, and III‐IV are highly malignant gliomas. The 2016 WHO classification was the first to introduce the classification of CNS tumors that integrate histological and genomic phenotypes.^3^, ^4^ The current therapeutic strategies to combat glioma, such as surgery, radiotherapy, and chemotherapy, have been improved. However, the overall survival of patients with glioma remains poor due to its infiltrative growth characteristics and chemotherapy resistance.^5^ Its potential mechanisms remain far from understood.^6^, ^7^ Therefore, it is of utmost importance to further explore its molecular mechanisms in order to select suitable predictive biomarkers.

Long noncoding RNAs (lncRNAs) are noncoding RNAs with transcripts longer than 200 nucleotides without the function of encoding proteins.^8^, ^9^, ^10^ Researchers found that lncRNAs play a critical regulatory role in dosage compensation effect, epigenetic regulation, cell cycle regulation, cell differentiation regulation, and many other life activities.^11^, ^12^ Unlike their shorter counterparts, such as microRNAs (miRNAs) and other smaller noncoding RNAs, lncRNAs can regulate downstream target genes by cis‐ and trans‐regulatory effects.^13^, ^14^ Recent studies reported that a growing number of lncRNAs can regulate the function of downstream genes through a “lncRNA‐miRNA‐mRNA” mode.^15^, ^16^, ^17^, ^18^ However, the function of many lncRNAs remains widely unknown.

LncRNA FOXD1‐AS1 (ENST00000514661.1/RP11‐79P5.2, FOXD1‐AS1) is the antisense transcript of the gene encoding for the protein Forkhead Box D1 (FOXD1). FOXD1 is highly expressed in the kidney and regulates the cellularity of the renal capsule, an important structure in normal kidney.^19^, ^20^ FOXD1 also exists in the brain and retina and is necessary for a normal retinal and optic chiasm development.^21^, ^22^ FOXD1 is also an oncogene as it promotes cell proliferation and chemotherapeutic drug resistance in breast cancer.^23^, ^24^ Moreover, FOXD1 silencing inhibits proliferation and migration in glioma cells.^25^ However, whether FOXD1‐AS1 is associated with glioma remains unknown.

In this study, we detected the expression, functional role, and underlying mechanism of FOXD1‐AS1 in glioma. The pathologic relevance of FOXD1‐AS1 in glioma growth and progression was characterized. Its expression and localization were analyzed by qPCR. In addition, further investigations into its function and mechanisms in glioma were performed using gain‐of‐function and loss‐of‐function studies, luciferase reporter assay, and chromatin isolation by RNA purification‐mass spectrometry (ChIRP‐MS). Our findings reveal that FOXD1‐AS1, a miR339/342 target, affected biological processes via protein eIF5a; thus, it might be considered as a novel emerging oncogenic biomarker and a potential target against glioma.

## 2 MATERIALS AND METHODS

### 2.1 Cell lines and culture

Human glioma cell lines U87, U251, U138, and Hs683 were received from the Institute of Biochemistry and Cell Biology of the Chinese Academy of Sciences and routinely cultured in Dulbecco's modified Eagle's medium supplemented with 10% fetal bovine serum (FBS). Cells were incubated at 37°C in a humidified atmosphere of 5% CO_2_. Cells were transfected using Lipofectamine® RNAiMAX Reagent according to the manufacturer's instructions. Nuclear/cytoplasmic extract was obtained using the Nuclear/Cytosol Extraction Kit (K266‐25, BioVision).

### 2.2 Xenograft model

BALB/c‐nu mice aged 5‐6 weeks were purchased from Slack Jingda Experimental Animal Co., Ltd. (ID: SCXK2011‐003). Mice arrived two weeks prior the experiment and were housed into microisolator cages during the entire course of the study. To obtain tumor xenografts, 100 *μ*L tumor cell suspension (5 ×  10^7^ cells/mL) was subcutaneously injected into the flank of nude mice. Tumor volume (V) was measured by a caliper and using the formula: V = 0. 5 × a × b^2^, where “a” is the maximum perpendicular diameter and “b” is the minimum perpendicular diameter.

### 2.3 Patient samples and real‐time quantitative PCR

Consecutive glioma patients who were newly diagnosed between 2007 and 2018 were recruited from the Affiliated Cancer Hospital of Xiangya, Central South University, and invited to participate in this institutional review board‐approved study. In majority of cases, the samples were histologically diagnosed according to the 2007 WHO classification, with a few samples depended on 2016 WHO classification (Table S1). Glioma tissues were collected after surgical resection, snap‐frozen in liquid nitrogen, and finally stored in the −80°C freezer before RNA extraction. Total RNA was extracted and reverse‐transcribed into cDNA. The qPCR solution used was 10 *μ*L 2 × SYBR premix ex‐taq, 2 *μ*L primers (F&R, 10 *μ*mol/L), 2 *μ*L cDNA, and ddH_2_O up to a final 20 *μ*L volume. Real‐time PCR was performed on a LightCycler 480 II plus Real‐Time PCR System. Primers used are shown in Table S2. Among them, mature miRNA was used as miRNA specific 5’ primer, while the 3’ primer for qPCR was supplied with the kit.

### 2.4 MTS assay

Cells were seeded into 96‐well culture plates (1,000 cells/well), and cell proliferation was measured at 24, 48, 72, and 96 hours after transfection through incubation in 100 *µ*L MTS reagent (Promega). Absorbance was measured at 490 nm using a BioTek®Eon (SynergyTM, HT). Background absorbance of the medium without cells was subtracted.

### 2.5 Colony formation assay

Cells were trypsinized and recultured into 6‐well plates with a density of 1000 cells/well. To form natural colonies, cells were allowed to grow for 12 days. Then, plates were washed with phosphate‐buffered saline (PBS) and colonies were stained with crystal violet. Stained colonies were photographed with a camera (Canon), and the number of colonies (>50 cells/colony) in each group was counted.

### 2.6 EdU incorporation assay

After transfection, cells were incubated with DMEM supplemented with 10 *μ*mol/L EdU according to the manufacturer's protocol (C10310, RiboBio). Then, cells were fixed with 4% formaldehyde at room temperature for 30 minutes. After staining, cells were observed under fluorescence microscope and images were recorded.

### 2.7 Apoptosis analysis

Cells were harvested, washed with PBS, and fixed in precooled 70% ethanol. Next, cells were suspended, filtered, stained with Annexin V/propidium iodide (PI) according to the manufacturer's protocol (AnnexinV‐FITC kit, Beyotime Biotechnology), and analyzed by flow cytometry.

### 2.8 Wound healing assay

Confluent monolayers were scratched using sterile pipette tips, trying to keep the scratch width consistent. The cell culture medium was removed, cells were washed with PBS to remove the cell debris, and serum‐free medium was added. Finally, images were taken for the analysis. Next, the plate was stored into the incubator and other images were taken at specific time intervals.

### 2.9 Western blot analysis

Proteins in total cell lysates were separated by sodium dodecyl sulfate polyacrylamide gel electrophoresis (SDS‐PAGE) and then transferred onto polyvinylidene fluoride membranes (PVDF, Millipore). PVDF was incubated with primary antibodies against eIF5a (ab32443; Abcam), CBL (ab52855; Abcam), or *β*‐actin (A5441; Sigma‐Aldrich LLC) overnight at 4°C. Subsequently, horseradish peroxidase‐conjugated goat anti‐rabbit IgG or goat anti‐mouse IgG was applied at room temperature for 2 hours. Membranes were analyzed by a ChemiDoc XRS + image analyzer (Bio‐Rad).

### 2.10 Luciferase activity assays

Cells were cotransfected with pmiR‐RB‐reporter vectors (WT/MUT) and miRNA mimics (Ribobio) using Lipofectamine 2000 (Invitrogen). After 48 hours, luciferase activity was measured using the Dual Luciferase Reporter Assay System (Promega). Subsequently, the luminescence meter was turned on, the delay was set at 2 seconds and the reading time at 10‐20 seconds. Results were obtained from three independent experiments, each with three technical replicates.

### 2.11 Chromatin isolation by RNA purification‐mass spectrometry (ChIRP‐MS) analysis

ChIRP‐MS is the best approach to find a target DNA/RNA/protein that is directly regulated by RNA. Protein‐RNA interactions were measured according to the manufacturer's protocol (Magnetic RNA‐Protein Pull‐Down Kit, Thermo). The enriched proteins were analyzed by liquid chromatography‐tandem mass spectrometry (LC‐MS/MS).

### 2.12 Statistical analysis

SPSS 19.0 software was used to perform the statistical analysis. The significance of the differences between groups was estimated by one‐way ANOVA or Student's *t* test. The association between target gene and clinical features was analyzed using chi‐square test. Data are presented as mean ± standard error of the mean (SEM). A value of *P* < 0.05 was considered statistically significant.

## 3 RESULTS

### 3.1 FOXD1‐AS1 is upregulated in glioma tissue

**Figure 1:**
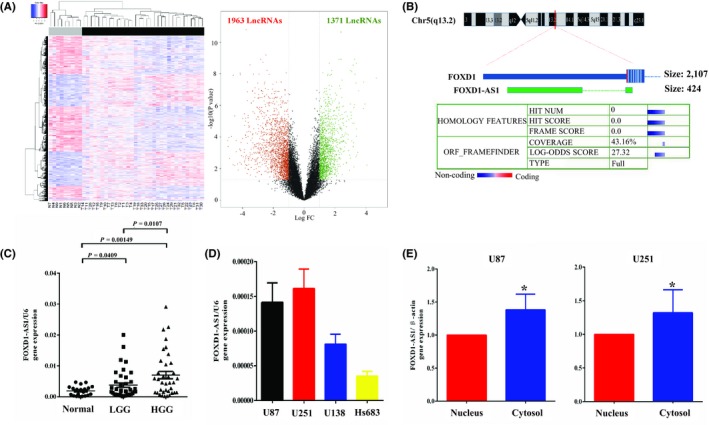
FOXD1‐AS1 is upregulated in glioma tissue. A, Microarray analysis showing 3334 lncRNAs with more than 2‐fold expression in glioma tissue compared with normal brain tissue. B, CPAT (Coding Potential Assessment Tool) indicated that FOXD1‐AS1 did not have any coding ability. C, FOXD1‐AS1 expression was significantly higher in the high‐grade glioma tissues compared with that in the normal brain tissues and low‐grade glioma tissues. D, FOXD1‐AS1 was higher in U87 and U251, while it was the lowest in Hs683 by qPCR. E, FOXD1‐AS1 was present in both the nucleus and the cytoplasm, although highly expressed in the cytoplasm. Data are presented as mean ± SEM *P < 0.05

Our research group has been searching for glioma biomarkers for a few years.^26^, ^27^, ^28^ To identify transcripts involved in glioma tumorigenesis, messenger RNA (mRNA), lncRNA, and miRNA expression profiles were determined by microarray analysis. Hierarchical clustering showed systematic variations in lncRNA expression levels between tumor and nontumor tissues, with 3334 lncRNAs expressed more than two times in glioma tissue compared to normal brain tissue (Figure 1A, Table S3). Among the lncRNAs, we were focusing our attention particularly on FOXD1‐AS1. CPAT (Coding Potential Assessment Tool, http://lilab.research.bcm.edu/cpat/index.php) results indicated FOXD1‐AS1 as a lncRNA without coding ability (Figure 1B). We carefully evaluated multiple housekeeping genes and selected those that had less variability across nontumor tissues used as controls and glioblastoma samples (Figure S1). Subsequently, qPCR was performed to further validate the expression of FOXD1‐AS1—one of the highly upregulated lncRNAs—in additional 75 glioma tissues and 20 normal brain tissues. Compared to normal brain tissues and low‐grade glioma tissues (LGG), FOXD1‐AS1 was significantly higher in high‐grade glioma (HGG) (Figure 1C). Seventy‐five glioma tissues were divided into two groups, based on the median expression level of all gliomas. The correlation between FOXD1‐AS1 expression and glioma clinicopathological characteristics was then measured and is summarized in Table 1.

**Table 1:** Correlation between FOXD1‐AS1 expression and glioma clinicopathological features in 75 patients

	N%	FOXD1‐AS1 expression levels		Ratio (High/Low)	*P*
		High expression	Low expression		
Sex					
Male	52 (69.3)	17	35	0.486	0.102
Female	23 (30.7)	9	14	0.643	
Age, y					
<45	48 (64.0)	17	31	0.548	0.0814
≥45	27 (36.0)	9	18	0.500	
Grade					
Low (I + II)	39 (52.0)	9	30	0.300	0.0107
High (III + IV)	36 (48.0)	17	19	0.895	

In addition, qPCR was used to assess FOXD1‐AS1 expression in 4 glioma cell lines and its spatial distribution. FOXD1‐AS1 was higher in U87 and U251, while showed the lowest expression in Hs683 (Figure 1D). Furthermore, FOXD1‐AS1 was localized in both the nucleus and the cytoplasm, although its localization was more abundant in the cytoplasm compared to the nucleus (Figure 1E).

### 3.2 FOXD1‐AS1 promotes glioma cell proliferation and tumor growth in vitro and in vivo

**Figure 2:**
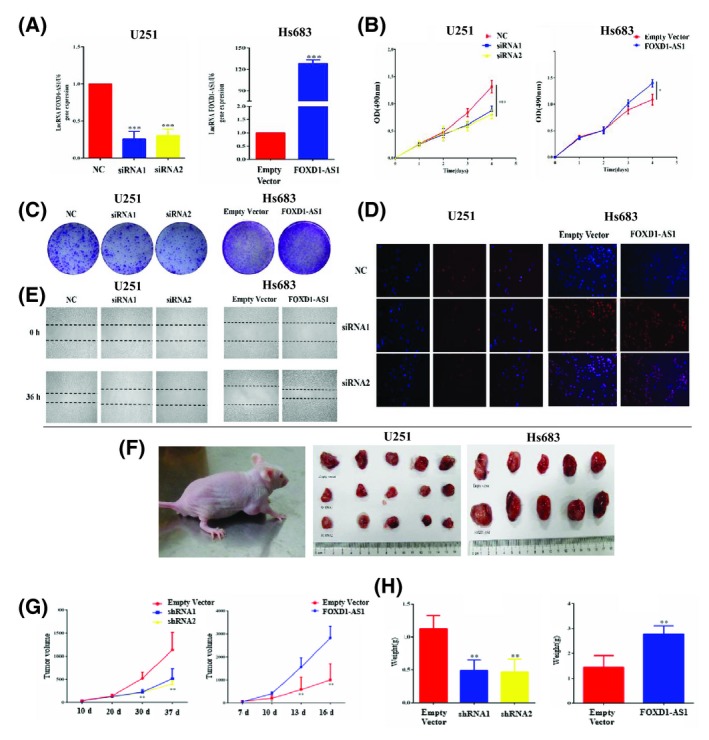
FOXD1‐AS1 promotes glioma cell proliferation and tumor growth in vitro and in vivo. A, siRNA and plasmid regulate FOXD1‐AS1 expression efficiently in human glioma cell lines. B, C, MTS and colony formation assay. FOXD1‐AS1 silencing dramatically reduced the proliferative ability of glioma cells. In contrast, FOXD1‐AS1 overexpression increased the proliferative ability of Hs683 cells. D, EdU assay. U251 and Hs683 glioma cell lines were treated with siRNA or plasmid alone, and EdU assay was performed. E, Wound healing assay. Wound healing assay indicates that FOXD1‐AS1 expression contributed to glioma cell migration. F‐H, Xenograft tumors obtained using FOXD1‐AS1 silenced cells resulted in a smaller mean volume and weight than controls. Tumors obtained using cells overexpressing FOXD1‐AS1 resulted in a larger mean volume and weight, with a more rapid development than tumors obtained from control cells. Data are presented as mean ± SEM *P < 0.05, **P < 0.01, and ***P < 0.001

To investigate the biological significance of FOXD1‐AS1 expression in the development and progression of glioma, gain‐of‐function and loss‐of‐function studies were performed in glioma cells. FOXD1‐AS1 was silenced in U251 cell line, while it was overexpressed in Hs683 cell line (Figure 2A). MTS and colony formation assay in vitro showed that FOXD1‐AS1 silencing dramatically reduced the proliferative ability of glioma cells. In contrast, FOXD1‐AS1 overexpression increased the proliferative ability of Hs683 cells compared to cells expressing the empty vector (Figure 2B,C). EdU assay results were consistent with the results obtained using the two assays mentioned above (Figure 2D), while wound healing assay indicated that FOXD1‐AS1 contributed to glioma cell migration (Figure 2E).

The effect of FOXD1‐AS1 expression on growth increase was confirmed by measuring in vivo tumor growth. Xenograft tumor growth from FOXD1‐AS1 silenced cells resulted in a smaller mean volume and weight compared to the negative control. Notably, tumor growth from cells overexpressing FOXD1‐AS1 showed larger mean volume and weight and developed more rapidly compared to tumors generated from cells transfected with scramble lncRNA used as control (Figure 2F‐H). These results demonstrated that FOXD1‐AS1 expression could promote glioma cell proliferation both in vitro and in vivo.

### 3.3 FOXD1‐AS1 is a direct target of miR339/342

**Figure 3:**
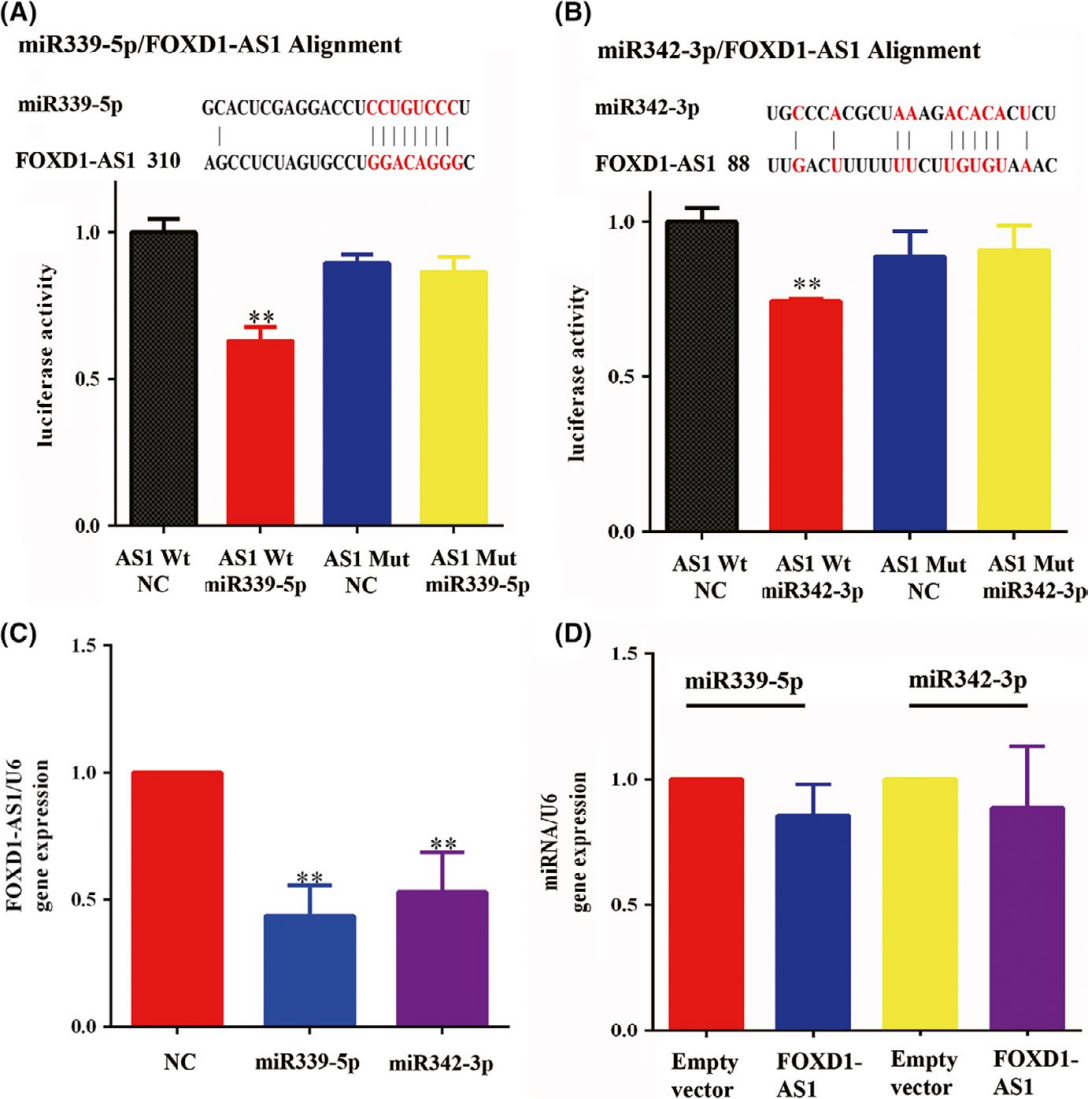
FOXD1‐AS1 is a direct target of miR339/342. A, B, Dual luciferase assay showed a significant decrease in luciferase activity after cotransfection of miR339/342 and wild‐type FOXD1‐AS1 expression vector, but not when a mutant FOXD1‐AS1 was used. C, miR339/342 overexpression significantly downregulated FOXD1‐AS1. D, FOXD1‐AS1 overexpression did not affect miR339/342 expression. Data are presented as mean ± SEM **P < 0.01, and ***P < 0.001

Interactions between lncRNAs and miRNAs provide an additional layer of control in gene regulation. By using RegRNA2 software (http://regrna2.mbc.nctu.edu.tw/), a set of miRNAs that putatively bind to FOXD1‐AS1 was found (Table S4). Among the predicted miRNAs, we were particularly interested in miR339/342 because of our previous results indicating these two as associated with FOXD1‐AS1. To further evaluate this association, luciferase activity assay was performed, and it showed a significant decrease in luciferase activity following the cotransfection of miR339/342 and the wild‐type FOXD1‐AS1 expression vector. This was not observed when cotransfection was performed using a mutant FOXD1‐AS1 expression vector (Figure 3A,B). The regulatory relationship between FOXD1‐AS1 and miR339/342 was further clarified. miR339/342 overexpression significantly downregulated FOXD1‐AS1 (Figure 3C), and FOXD1‐AS1 overexpression did not affect miR339/342 expression, suggesting that FOXD1‐AS1 is a direct negative target of miR339/342 (Figure 3D). Collectively, these data demonstrated miR339/342 binding to FOXD1‐AS1 and it might serve as a negative upstream regulator of FOXD1‐AS1.

### 3.4 miR339/342 inhibits cell proliferation by regulating FOXD1‐AS1

**Figure 4:**
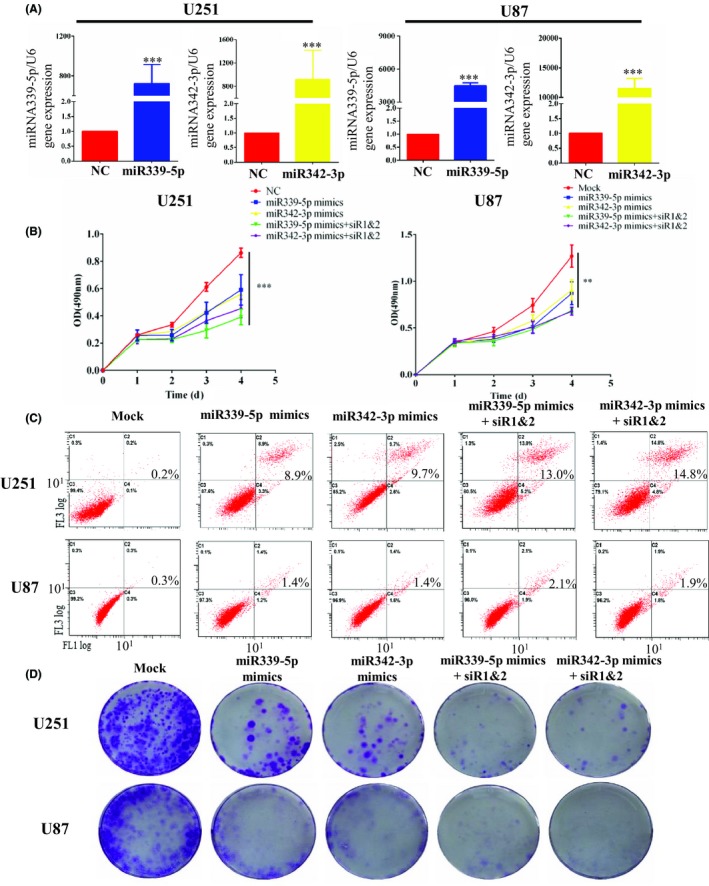
miR339/342 inhibit cell proliferation by regulating FOXD1‐AS1. A, miRNA mimics enhanced miR339/342 expression efficiently in human glioma cell lines. B, MTS assay. The result showed that miR339/342 overexpression dramatically reduced proliferation of glioma cells, compared with the mock group. The mixture group also prevented glioma cell growth. Interestingly, no change in proliferation was observed between miR339/342 group and mixture group. C, Flow cytometric analysis also showed that the miR339/342 mimics and mixture group prevented glioma cell growth compared to the mock‐treated group. D, Colony formation assay. Long‐term colony formation assay revealed that the mixture group dramatically reduced colony formation ability of glioma cells compared to the miR339/342 mimics group. Data are presented as mean ± SEM **P < 0.01, and ***P < 0.001

To further confirm the relationship between miR339/342 and FOXD1‐AS1, cells were transfected with siRNA targeting FOXD1‐AS1 and miR339/342 mimics to observe the influence on cell proliferation. miRNA mimics enhanced miR339/342 expression efficiently in human glioma cell lines (Figure 4A). MTS results showed that miR339/342 overexpression dramatically reduced the proliferative ability of glioma cells compared to the mock‐treated group. The mixture of siRNA‐ FOXD1‐AS1 and miR339/342 mimics (mixture group) also prevented glioma cell growth. Interestingly, no change in cell growth was observed between miR339/342 mimics group and mixture group (Figure 4B). Flow cytometric analysis also showed that the miR339/342 mimics and mixture group prevented glioma cell growth compared to the mock‐treated group (Figure 4C). Importantly, long‐term colony formation assay revealed that the mixture group dramatically reduced the colony formation ability of glioma cells compared to the miR339/342 mimics group (Figure 4E). These results suggested that miR339/342 inhibited cell proliferation by regulating FOXD1‐AS1.

### 3.5 FOXD1‐AS1 affects biological processes via protein eIF5a

**Figure 5:**
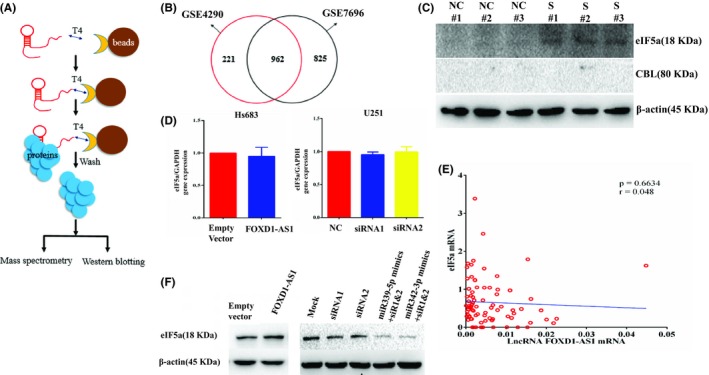
FOXD1‐AS1 affects biological processes via protein eIF5a. A, ChIRP‐MS diagram. B, Intersection of the identified DEGs in GSE4290 and GSE7696, revealing 962 DEGs in GBM. C, Western blot results showing that eIF5a could bind to FOXD1‐AS1, whereas the corresponding protein CBL could not. D, eIF5a mRNA was not changed when the expression of FOXD1‐AS1 was adjusted in glioma cells. E, No correlation was observed between eIF5a mRNA and FOXD1‐AS1 in glioma tissue. F, Western blot was used to investigate the relationship between FOXD1‐AS1 and eIF5a at a protein level. The result showed that eIF5a protein was positively proportional to FOXD1‐AS1 protein

To gain insights into proteins bound to FOXD1‐AS1, ChIRP‐MS and bioinformatics analyses were used, involving also ChIRP‐Seq, a method using DNA oligonucleotides to capture lncRNAs and their genomic DNA binding sites.^29^ ChIRP‐MS was optimized to identify lncRNA‐associated proteins (Figure 5A), and the results showed a number of 130 proteins that can bind to FOXD1‐AS1. To improve accuracy, bioinformatics analysis was performed. GSE4290 and GSE7696 data were used to evaluate differentially expressed genes (DEGs) by bioinformatics analysis. Comparison of identified DEGs from the two profiles revealed 962 DEGs in glioblastoma (Figure 5B, Figure S2). Further comparison of the above data revealed 37 differentially expressed proteins (Table S5).

For further verification, Western blot results showed that eIF5a could bind to FOXD1‐AS1, whereas the corresponding protein CBL could not bind (Figure 5C). qPCR and Western blot were used to investigate the relationship between FOXD1‐AS1 and eIF5a. The result showed that eIF5a mRNA was not changed when the expression of FOXD1‐AS1 was adjusted in Hs683 and U251 glioma cell lines (Figure 5D). In addition, no correlation was found between eIF5a mRNA and FOXD1‐AS1 in clinical glioma samples (Figure 5E). Importantly, Western blot showed that eIF5a protein was positively proportional to FOXD1‐AS1 at a posttranscriptional level (Figure 5F). Altogether, these data showed that FOXD1‐AS1 affected biological processes via protein eIF5a.

## 4 DISCUSSION

lncRNAs are classified according to their genomic locations and context.^30^ Some studies showed that different lncRNAs have different roles, and some act as transcriptional regulators, altering gene transcription.^31^ With the rapid development of epigenetics in recent years, plenty of lncRNAs that are aberrantly expressed in glioma can affect cancer progression. Moreover, vital roles in glioma are known. In this study, differences between lncRNA expression profiles in glioma tissue and nontumor tissues were assessed via lncRNA expression by microarray experiments. Notably, 1,371 lncRNAs were found upregulated, while 1,963 lncRNAs were downregulated in our microarray results. From these data, the focus was on upregulated lncRNAs as this set can be used more readily than downregulated lncRNAs to find appropriate early diagnostic markers or therapeutic targets. Among them, FOXD1‐AS1 is the antisense transcript of the gene encoding for the protein Forkhead Box D1 (FOXD1). Until now, no study explored the role of FOXD1‐AS1 in glioma. The current study found that FOXD1‐AS1 was upregulated and directly correlated with the glioma grade. In addition, our data showed that FOXD1‐AS1 promoted glioma cell proliferation and tumor growth in vitro and in vivo. Therefore, our results indicated that FOXD1‐AS1 acted as an oncogene in glioma.

Both lncRNAs and miRNAs play dynamic roles in transcriptional and translational regulation, and the interaction between them is called competitive endogenous RNA (ceRNAs).^32^, ^33^ It provides a new theoretical basis for studying the pathogenesis of tumor and other diseases. Interactions between lncRNAs and miRNAs have recently been reported, and the interest in this subject is increased. For example, CHRF serves as an endogenous “sponge” for miR489 to regulate Myd88 expression and hypertrophy.^34^ The inhibition of lncRNA Gas5 is related to the upregulated miR222.^35^ Identification of miRNAs is important for exploring the molecular mechanisms underlying FOXD1‐AS1 function. In this work, a strong evidence (luciferase assay) indicated that miR339/342 bound directly to FOXD1‐AS1. It is interesting to notice that overexpression of FOXD1‐AS1 could not promote miR339/342 expression, indicating the existence of only a single‐line regulation mode of FOXD1‐AS1 in this pathway with miR339/342.

It has been confirmed that many lncRNAs could regulate the expression of some proteins through the interaction with RNA‐binding proteins.^36^ Our study indicated that FOXD1‐AS1 bound to protein eIF5a in some way. Interestingly, regulation of FOXD1‐AS1 in glioma cells did not significantly affect eIF5a mRNA expression. However, FOXD1‐AS1 was able to affect eIF5a protein expression, indicating that FOXD1‐AS1 regulated eIF5a expression at a posttranscriptional level. eIF5a functions as a nucleocytoplasmic shuttle protein. It has been proposed that eIF5a is an essential regulator of the nuclear export of some specific RNAs.^37^ These findings suggest that the FOXD1‐AS1/eIF5a axis might be involved in the initiation and development of glioma.

**Figure 6:**
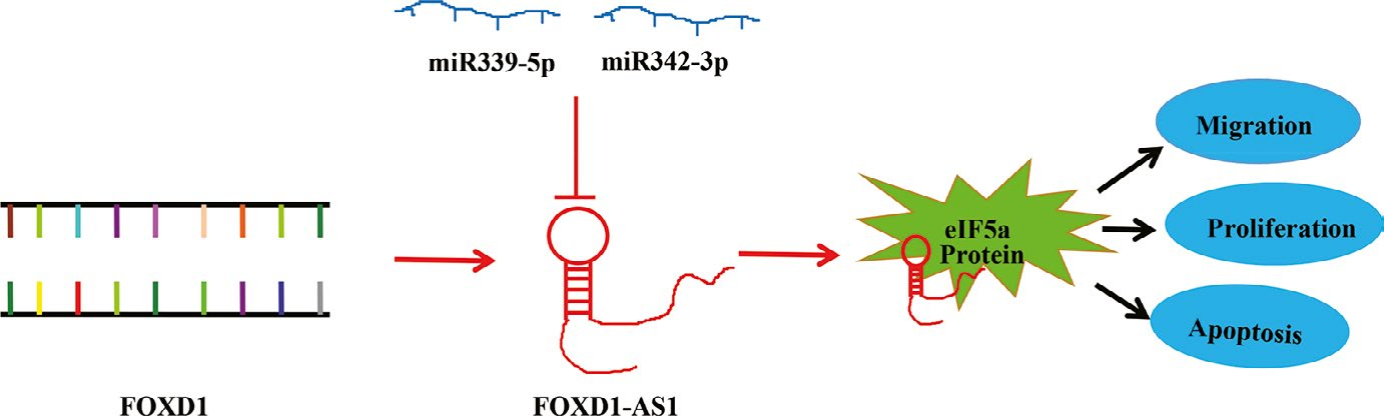
A summary of this study. FOXD1‐AS1, a miR339/342 target, might function as an oncogene, facilitating tumor cell proliferation and inhibiting apoptosis via targeting eIF5a in glioma

In conclusion, our results showed that FOXD1‐AS1, a miR339/342 target, might function as an oncogene to facilitate tumor cell proliferation and inhibited apoptosis via targeting protein eIF5a in glioma. A summary of our study is provided in Figure 6. These findings indicated that FOXD1‐AS1 might be a critical molecule in tumor progression and might be considered as an effective target in glioma therapy.

## CONFLICTS OF INTEREST

No potential conflicts of interest were disclosed.
