# Supplementary figures and images for: LncRNA FOXD1‐AS1 acts as a potential oncogenic biomarker in glioma
Source: CNS Neurosci Ther. 2019 May 17;26(1):66–75. doi: 10.1111/cns.13152 (PMC6930828; doi:10.1111/cns.13152)

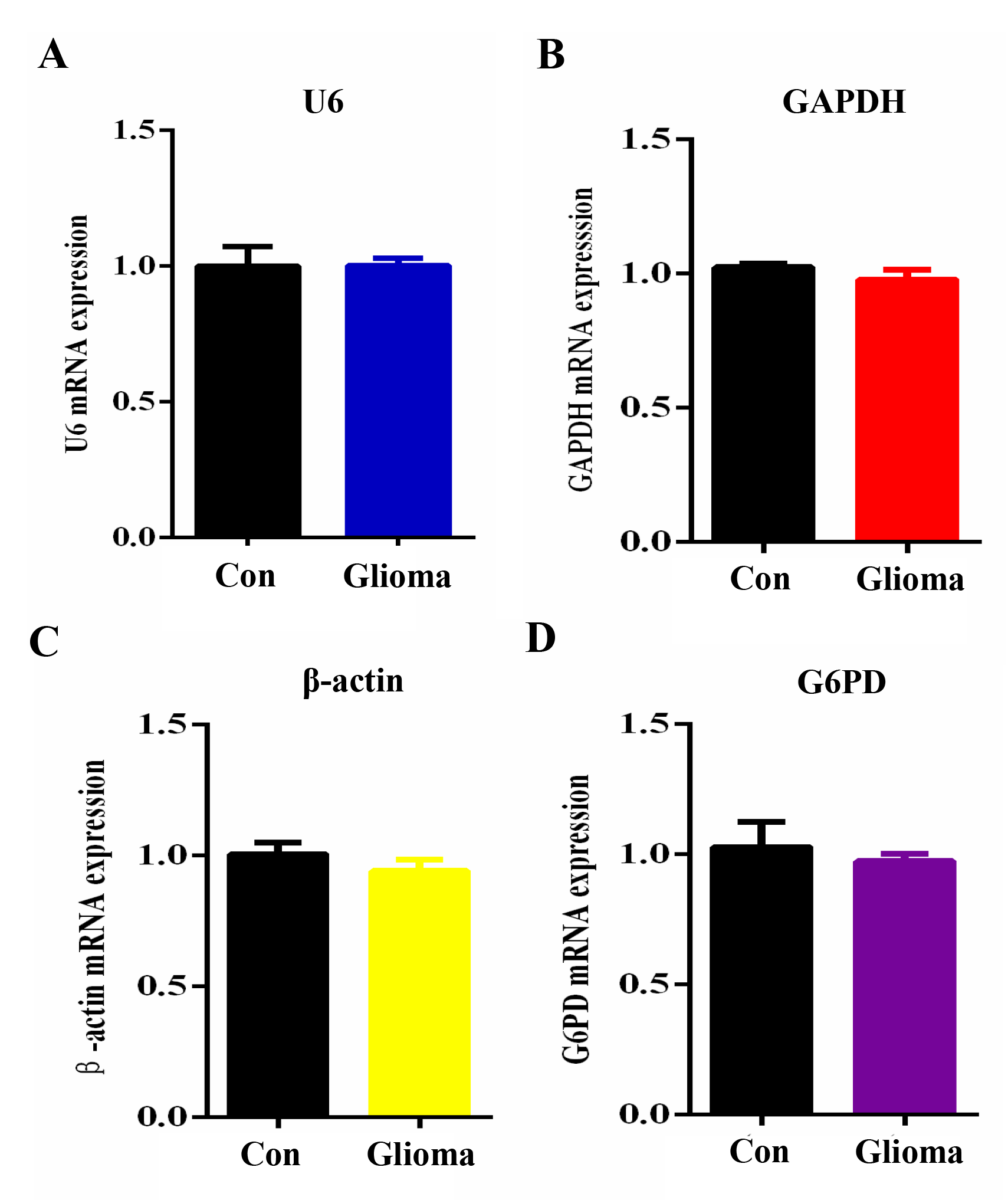

Supplement: Supplementary file 1 [file CNS-26-66-s001.tif]

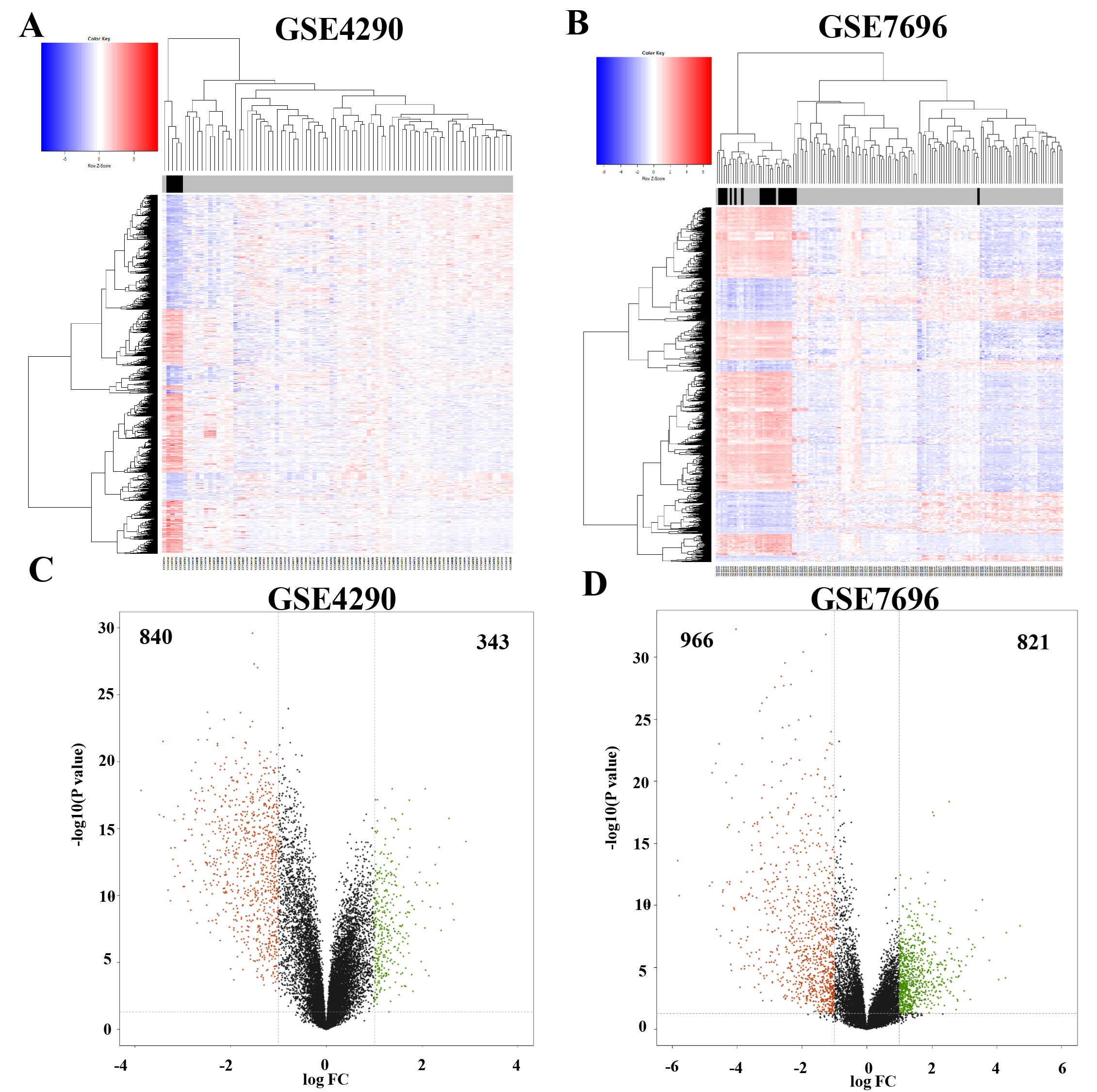

Supplement: Supplementary file 2 [file CNS-26-66-s002.tif]
