# Supplementary material for: LncRNA FOXD1‐AS1 acts as a potential oncogenic biomarker in glioma
Source: CNS Neurosci Ther. 2019 May 17;26(1):66–75. doi: 10.1111/cns.13152 (PMC6930828; doi:10.1111/cns.13152)
Supplement: Supplementary file 4 [file CNS-26-66-s004.doc]

**Table S2.** Primer sequences used for real-time PCR.

| Gene | Sequence | Base |
| --- | --- | --- |
| FOXD1-AS1-F | TTTTAACGCCTGGACCTGAGAAT | 23 |
| FOXD1-AS1-R | GTTAATAACGCTATGCTACAGCC | 23 |
| eIF5a -F | GGACGACCATGCAAAATAGTGG | 22 |
| eIF5a -R | TGCCCGTGAAAATATCAATTCCA | 23 |
| U6-F | CTCGCTTCGGCAGCACA | 17 |
| U6-R | AACGCTTCACGAATTTGCGT | 20 |
| GAPDH-F | GAGTCAACGGATTTGGTCGT | 20 |
| GAPDH -R | TTGATTTTGGAGGGATCTCG | 20 |
