# Supplementary material for: LncRNA FOXD1‐AS1 acts as a potential oncogenic biomarker in glioma
Source: CNS Neurosci Ther. 2019 May 17;26(1):66–75. doi: 10.1111/cns.13152 (PMC6930828; doi:10.1111/cns.13152)
Supplement: Supplementary file 5 [file CNS-26-66-s005.docx]

**Table S3** Up-regulated lncRNAs in glioma chips (Top50)

| NO. | lncRNA | Chrosome | Start | End | gene | p | FC |
| --- | --- | --- | --- | --- | --- | --- | --- |
| 1 | TCONS_00010961 | 5 | 72542336 | 72570644 | XLOC_004881 | 1.16E-11 | 26.15368 |
| 2 | hox-HOXA7-15 | 7 | 26967529 | 26967845 | hox-HOXA7-15 | 0.001143718 | 15.69416 |
| 3 | HIT000332651 | 19 | 6680187 | 6720573 | HIX0040474 | 1.11E-08 | 13.95972 |
| 4 | ENST00000428939.3 | 7 | 27138457 | 27139758 | ENSG00000233429.5 | 0.0000514 | 11.27222 |
| 5 | nc-HOXA5-68 | 7 | 26956776 | 26957425 | nc-HOXA5-68 | 0.000131368 | 10.34163 |
| 6 | ENST00000443546.1 | 6 | 10414810 | 10415955 | ENSG00000229950.1 | 3.25E-09 | 9.674631 |
| 7 | ENST00000522197.1 | 8 | 122652141 | 122654957 | ENSG00000248690.2 | 0.000000347 | 9.024598 |
| 8 | hox-HOXA7-17 | 7 | 26969046 | 26969505 | hox-HOXA7-17 | 0.001542161 | 8.896306 |
| 9 | TCONS_00025723 | 17 | 59470820 | 59474207 | XLOC_012542 | 1.68E-12 | 8.857987 |
| 10 | NR_038435.1 | 2 | 176999568 | 177001826 | HOXD-AS2 | 0.00000712 | 8.264662 |
| 11 | ENST00000555772.1 | 15 | 94607597 | 94614049 | ENSG00000258754.2 | 0.001897944 | 8.003608 |
| 12 | ENST00000455011.1 | 6 | 141167094 | 141174061 | ENSG00000234147.1 | 0.000182791 | 7.971706 |
| 13 | ENST00000553909.1 | 14 | 21152745 | 21168735 | ENSG00000259171.1 | 0.00000402 | 7.151776 |
| 14 | ENST00000416008.1 | 2 | 105027658 | 105032070 | ENSG00000227157.1 | 0.00000408 | 7.001801 |
| 15 | ENST00000586661.1 | 17 | 74149792 | 74150724 | ENSG00000267128.1 | 0.000134315 | 6.889277 |
| 16 | ENST00000506791.1 | 5 | 43018584 | 43024336 | ENSG00000251131.1 | 0.000000258 | 6.598799 |
| 17 | ENST00000453229.1 | 1 | 63154152 | 63176365 | ENSG00000235545.1 | 0.0000169 | 6.228354 |
| 18 | ENST00000562669.1 | 18 | 65149027 | 65152203 | ENSG00000260578.1 | 0.0000052 | 6.159031 |
| 19 | **ENST00000514661.1** | 5 | 72742183 | 72742811 | ENSG00000247993.2 | 0.0000123 | 6.080129 |
| 20 | ENST00000455981.1 | 9 | 132044736 | 132048007 | ENSG00000224307.1 | 2.81E-08 | 6.048066 |
| 21 | ENST00000564401.1 | 15 | 56686518 | 56688983 | ENSG00000259941.1 | 4.79E-09 | 5.865641 |
| 22 | NR_038303.1 | 4 | 79567147 | 79605655 | LOC100505702 | 0.00000266 | 5.842565 |
| 23 | ENST00000456535.1 | 3 | 186194515 | 186196159 | ENSG00000226859.1 | 0.0000566 | 5.750301 |
| 24 | ENST00000514586.1 | 5 | 38783681 | 38792856 | ENSG00000250629.1 | 0.0000136 | 5.690942 |
| 25 | TCONS_00008049 | 4 | 38749926 | 38754586 | XLOC_003514 | 0.000000775 | 5.665113 |
| 26 | ENST00000438047.1 | 7 | 157647276 | 157658784 | ENSG00000233038.1 | 0.0000139 | 5.608554 |
| 27 | ENST00000523301.1 | 5 | 158527629 | 158534225 | ENSG00000245812.2 | 4.65E-09 | 5.597222 |
| 28 | ENST00000413039.1 | 6 | 32811909 | 32813763 | ENSG00000204261.4 | 0.000000696 | 5.5811 |
| 29 | ENST00000495081.2 | 3 | 179115495 | 179116471 | ENSG00000242539.2 | 0.000000412 | 5.513768 |
| 30 | ENST00000431376.1 | 2 | 28856147 | 28887406 | ENSG00000230730.1 | 0.00000206 | 5.510478 |
| 31 | ENST00000509088.1 | 4 | 79892901 | 80229698 | ENSG00000249307.1 | 0.005691871 | 5.462584 |
| 32 | ENST00000566551.1 | 1 | 23607801 | 23613245 | ENSG00000261326.1 | 0.0000265 | 5.459302 |
| 33 | ENST00000445551.1 | 1 | 47897804 | 47900313 | ENSG00000237424.1 | 0.00000787 | 5.45756 |
| 34 | TCONS_00023604 | 15 | 32474849 | 32500363 | XLOC_011414 | 0.00000603 | 5.455119 |
| 35 | ENST00000436616.1 | 2 | 179278725 | 179298716 | ENSG00000223960.1 | 2.07E-11 | 5.41692 |
| 36 | ENST00000416329.1 | 1 | 101529166 | 101549039 | ENSG00000233184.1 | 0.000000652 | 5.398372 |
| 37 | ENST00000555294.1 | 14 | 23452106 | 23467632 | ENSG00000258457.1 | 0.000000408 | 5.377499 |
| 38 | ENST00000526206.1 | 11 | 86414140 | 86425134 | ENSG00000254731.1 | 0.00000039 | 5.366095 |
| 39 | ENST00000513899.1 | 5 | 82837295 | 82877139 | ENSG00000249835.2 | 0.000000603 | 5.351533 |
| 40 | ENST00000588689.1 | 6 | 112475992 | 112476934 | ENSG00000237234.2 | 4.95E-08 | 5.348828 |
| 41 | ENST00000415809.1 | 11 | 3875547 | 3876739 | ENSG00000228661.1 | 1.18E-09 | 5.34343 |
| 42 | ENST00000579138.1 | 17 | 65028524 | 65029406 | ENSG00000265664.1 | 0.0000062 | 5.323791 |
| 43 | XR_108682.1 | 6 | 19803946 | 19804910 | LOC100506885 | 0.0000163 | 5.271645 |
| 44 | HIT000071394 | 19 | 49340353 | 49340557 | HIX0040211 | 0.00000521 | 5.25017 |
| 45 | uc021rel.1 | 12 | 117467713 | 117469233 | AK055849 | 7.18E-16 | 5.242401 |
| 46 | ENST00000456355.1 | 10 | 17275323 | 17276832 | ENSG00000234961.1 | 0.000133494 | 5.16529 |
| 47 | uc003ttf.3 | 7 | 64141510 | 64147264 | BC053669 | 0.00000167 | 5.135876 |
| 48 | ENST00000600008.1 | 19 | 17516632 | 17525277 | ENSG00000269640.1 | 0.00000365 | 5.132848 |
| 49 | ENST00000376797.3 | 6 | 29968787 | 30028921 | ENSG00000204623.4 | 0.00000047 | 5.124056 |
| 50 | ENST00000460407.1 | 3 | 152557178 | 152559228 | ENSG00000241732.1 | 0.0000436 | 5.055701 |
