# Supplementary material for: LncRNA FOXD1‐AS1 acts as a potential oncogenic biomarker in glioma
Source: CNS Neurosci Ther. 2019 May 17;26(1):66–75. doi: 10.1111/cns.13152 (PMC6930828; doi:10.1111/cns.13152)
Supplement: Supplementary file 7 [file CNS-26-66-s007.docx]

**Table S5.** proteins that putatively bind to lncRNA FOXD1-AS1

| NO. | Accession | Pro name | Score | Coverage | # Proteins | # Peptides | # PSMs | # AAs | MW [kDa] | calc. pI |
| --- | --- | --- | --- | --- | --- | --- | --- | --- | --- | --- |
| 1 | Q09666 | AHNAK | 16.06 | 6.32 | 5 | 5 | 8 | 5890 | 628.7 | 6.15 |
| 2 | S4R471 | AMBP | 14.44 | 6.22 | 2 | 1 | 4 | 193 | 21.4 | 7.25 |
| 3 | H0YMM1 | ANXA1 | 1.66 | 12.75 | 27 | 2 | 2 | 149 | 16.4 | 5.91 |
| 4 | A0JLQ0 | AZGP1 | 7.39 | 7.55 | 3 | 1 | 2 | 159 | 18.7 | 8.97 |
| 5 | E5RG81 | CA1 | 5.78 | 11.02 | 5 | 1 | 2 | 127 | 14.0 | 9.16 |
| 6 | P22681 | CBL | 20.56 | 3.31 | 1 | 1 | 6 | 906 | 99.6 | 6.54 |
| 7 | O43866 | CD5L | 5.66 | 6.05 | 1 | 1 | 2 | 347 | 38.1 | 5.47 |
| 8 | L8E9M3 | CKAP2L | 2.92 | 31.11 | 1 | 1 | 1 | 45 | 5.1 | 10.02 |
| 9 | C9JAU3 | COBLL1 | 1.92 | 5.73 | 9 | 1 | 1 | 157 | 17.5 | 9.23 |
| 10 | J3QT83 | COL14A1 | 2.22 | 1.30 | 3 | 1 | 1 | 843 | 92.6 | 4.77 |
| 11 | D3DTX7 | COL1A1 | 2.84 | 2.03 | 1 | 1 | 1 | 885 | 84.7 | 6.24 |
| 12 | A8MWQ5 | COL25A1 | 0.00 | 6.05 | 2 | 1 | 1 | 645 | 63.3 | 7.94 |
| 13 | I3L397 | **eIF5a** | 21.2 | 9.48 | 4 | 2 | 1 | 146 | 16.0 | 5.00 |
| 14 | B7ZLE5 | FN1 | 41.82 | 4.29 | 20 | 6 | 10 | 2240 | 246.5 | 6.06 |
| 15 | Q6PJY1 | FUBP1 | 3.10 | 4.28 | 7 | 1 | 1 | 304 | 32.0 | 8.84 |
| 16 | H0Y6A7 | GDAP1 | 0.00 | 4.78 | 7 | 1 | 1 | 251 | 28.9 | 6.29 |
| 17 | Q9Y5P6 | GMPPB | 0.00 | 2.78 | 2 | 1 | 1 | 360 | 39.8 | 6.61 |
| 18 | H0YIY4 | GPHN | 1.62 | 13.33 | 1 | 1 | 1 | 60 | 6.9 | 10.24 |
| 19 | P80108 | GPLD1 | 1.83 | 1.55 | 1 | 1 | 1 | 840 | 92.3 | 6.37 |
| 20 | Q6B823 | Histone H4 | 5.98 | 18.60 | 3 | 1 | 3 | 43 | 4.9 | 10.92 |
| 21 | I3L239 | HSP75 | 2.74 | 6.51 | 18 | 1 | 1 | 215 | 24.9 | 9.31 |
| 22 | E9PN25 | HSPA8 | 1.96 | 8.33 | 39 | 1 | 1 | 132 | 14.6 | 6.55 |
| 23 | C9J3N8 | HSPB1 | 3.18 | 45.95 | 3 | 1 | 1 | 37 | 3.9 | 5.02 |
| 24 | Q0VAA2 | LRRC74A | 0.00 | 3.89 | 1 | 1 | 1 | 488 | 54.5 | 5.34 |
| 25 | G1UI26 | Nbas | 2.07 | 1.35 | 2 | 1 | 1 | 1561 | 176.2 | 5.76 |
| 26 | I3L425 | PEDF | 3.25 | 16.09 | 6 | 1 | 1 | 87 | 9.1 | 4.51 |
| 27 | B4DNK4 | PKM | 4.35 | 4.38 | 16 | 2 | 2 | 457 | 49.9 | 7.83 |
| 28 | A0A0A0MSI0 | PRDX1 | 2.03 | 12.28 | 4 | 2 | 2 | 171 | 19.0 | 6.92 |
| 29 | Q5JSZ5 | PRRC2B | 0.00 | 1.48 | 1 | 1 | 1 | 2229 | 242.8 | 8.34 |
| 30 | A0A087WUW5 | PTBP1 | 2.08 | 9.88 | 5 | 1 | 1 | 81 | 9.0 | 9.99 |
| 31 | Q5W0S5 | Rad23b | 2.10 | 6.85 | 4 | 1 | 1 | 146 | 15.2 | 6.77 |
| 32 | P61225 | RAP2B | 0.00 | 11.48 | 1 | 1 | 1 | 183 | 20.5 | 4.81 |
| 33 | A0A0C4DGV7 | RBP4 | 63.31 | 43.22 | 3 | 5 | 14 | 199 | 22.9 | 5.72 |
| 34 | D6RHD2 | SPRY1 | 3.23 | 8.13 | 2 | 1 | 1 | 209 | 22.9 | 8.82 |
| 35 | A0AVP6 | UTS2 | 0.00 | 8.51 | 4 | 1 | 1 | 94 | 11.0 | 7.88 |
| 36 | Q8N2Y6 | ZFHX3 | 0.00 | 4.06 | 2 | 1 | 1 | 886 | 98.5 | 6.90 |
| 37 | K7EM32 | Znf581 | 2.20 | 17.99 | 2 | 1 | 1 | 139 | 15.2 | 7.99 |
